# Downregulation of Organ‐Derived Activin A Attenuates Muscle Atrophy and Intramuscular Fat Infiltration in Cancer Cachexia Mice

**DOI:** 10.1002/jcsm.70237

**Published:** 2026-03-11

**Authors:** Cui Wang, Lin Gao, Rui Xue, Jia Su, Honghui Li, Wei Yang, Yan Tang, Zhihang Su, Shasha Min, Changyong Tang, Yuqi Zhu, Bo Mu, John R. Speakman, Xina Xie, Zesong Li

**Affiliations:** ^1^ Institute of Basic Medicine, School of Basic Medicine and Forensic Medicine North Sichuan Medical College Nanchong Sichuan China; ^2^ Department of Medical Research Center Yuebei People's Hospital Affiliated to Shantou University Medical College Shaoguan China; ^3^ Guangdong Provincial Key Laboratory of Systems Biology and Synthetic Biology for Urogenital Tumors, Shenzhen Key Laboratory of Genitourinary Tumor, Department of Urology, Shenzhen Institute of Translational Medicine, The First Affiliated Hospital of Shenzhen University Shenzhen Second People's Hospital Shenzhen China; ^4^ Shenzhen Key Laboratory of Metabolic Health, Center for Energy Metabolism and Reproduction Shenzhen Institute of Advanced Technology, Chinese Academy of Sciences Shenzhen China; ^5^ Department of Nephrology The First Affiliated Hospital of Shenzhen University, Shenzhen Second People's Hospital Shenzhen China; ^6^ Department of Critical Care Medicine, Longgang Central Hospital (Shenzhen Clinical College) Guangzhou University of Chinese Medicine Shenzhen Guangdong China; ^7^ Department of Neurology Third Affiliated Hospital of Sun Yat‐Sen University Guangzhou Guangdong Province China

**Keywords:** Activin A, cancer cachexia, heart and kidney, intramuscular fat infiltration, muscle atrophy

## Abstract

**Background:**

Cancer cachexia is a multifactorial wasting syndrome marked by profound skeletal muscle loss. Tumours can release high levels of Activin A (ActA), which activates the ubiquitin‐proteasome pathway (UPP) and drives muscle wasting. Systemic blockade of the ActA pathway is associated with inflammatory adverse effects, and tumour‐restricted targeting alone often fails to reverse cachexia. We asked whether ActA produced by host (nontumour) organs contributes to circulating ActA and muscle wasting.

**Methods:**

We profiled ActA across tissues and in serum in Lewis lung carcinoma (LLC) cancer cachexia mice to generate an organ‐wide expression map. Functional studies were then performed using adeno‐associated‐virus (AAV)‐knockdown in the heart (cTnT/hTCF21 promoters) and kidney (CMV promoter), followed by cachexia induction. Body weight (BW), food intake, skeletal muscle mass, muscle function and muscle histomorphology were assessed. Mitochondrial ultrastructure and lipid metabolic pathways in muscle and adipose tissue were also examined.

**Results:**

LLC cachexia mice exhibited significant reductions in body weight (**−**6.0%, *p* < 0.05), food intake (−9.9%, *p* < 0.05), quadriceps mass (−15.3%, *p* < 0.05) and grip strength (−13.0%, *p* < 0.0001) compared with non–tumour‐bearing (NTB) mice (*n* = 6–12/group). ActA expression was markedly increased in the host organs, particularly in the kidney (2.8‐fold vs. NTB, *p* < 0.001) and heart (2.7‐fold vs. NTB, *p* < 0.05) (*n* = 10/group). Compared with the sh‐NC, organ‐targeted ActA knockdown restored body weight (+6.1%, *p* < 0.05) and food intake (+8.4%**,** *p* < 0.05), increased quadriceps mass (+17.2%, *p* < 0.05) and grip strength (+10.7%, *p* < 0.01), reduced intramuscular fat infiltration and attenuated UPP signalling (*n* = 8–16/group). These effects were accompanied by increased expression of the mitochondrial fatty‐acid oxidation regulator carnitine palmitoyltransferase 1B (CPT1B) (+42.3% of mRNA level; +30.9% of protein level; both *p* < 0.05) and CPT2 (+57.7% of mRNA level, *p* < 0.05), improved mitochondrial ultrastructure and partial restoration of adipose mass.

**Conclusions:**

Simultaneous downregulation of Activin A in the kidney and heart attenuates skeletal muscle atrophy and intramuscular adipogenesis, improves muscle mass and function and mitigates adipose tissue mass loss in cancer cachexia mice. These findings identify heart‐ and kidney‐derived Activin A as a key driver of cachexia, which acts through a combinatorial effect rather than an isolated contribution from either one alone, highlighting its potential as a therapeutic target.

**Abbreviations**
AAV: adeno‐associated‐virus
ACC1: acetyl‐CoA carboxylase 1
ACOX1: acyl‐CoA oxidase 1
ActA: Activin A
ACVR2A/2B: activin receptor type 2A/2B
ANCOVA: analysis of covariance
ANOVA: analysis of variance
AOD: average optical density
ATCC: American Type Culture Collection
BNP: B‐type Natriuretic Peptide.
BW: body weight
CPT1B/2: carnitine palmitoyltransferase 1B/2
CSA: cross‐sectional areas
cTnT: cardiac troponin T
DAB: diaminobenzidine
eWAT: epididymal white adipose tissue.
FAO: fatty acid oxidation
FAS: fatty acid synthase
GEO: Gene Expression Omnibus
H&E: haematoxylin and eosin
IHC: immunohistochemical staining
IMF: intramuscular fat
LLC: Lewis lung carcinoma
NC: negative control
NTB: non–tumour‐bearing
ORO: oil red O
PAS: periodic acid–Schiff
SCD1: stearoyl‐CoA desaturase 1
scRNA‐seq: single‐cell transcriptome sequencing
snRNA‐seq: single‐nucleus transcriptome sequencing
sWAT: subcutaneous white adipose tissue
TBP: TATA‐box binding protein
TEM: transmission electron microscopy
UA: uric acid
UPP: ubiquitin proteasome pathway

## 1 Introduction

Cancer is a leading cause of death worldwide. The most recent WHO/IARC estimates report 9.7 million cancer deaths in 2022 (20 million new cases) [1], with the global burden continuing to rise. In advanced diseases, up to 80% of patients develop cachexia, which accounts for roughly 20%–40% of cancer‐related deaths [2, 3]. Cancer cachexia was defined as a multifactorial syndrome featured with an ongoing loss of skeletal muscle mass (with or without loss of fat mass) that cannot be fully reversed by conventional nutritional support and triggers progressive functional impairment [2, 4, 5]. Cancer cachexia has a negative energy balance [6], and multiple organs are involved in cancer cachexia, such as kidney, heart, liver and so on [7, 8]. The main symptoms of cancer cachexia include dramatic involuntary body weight loss, inflammation, loss of appetite, anaemia and insulin resistance [4]. Although we have made great efforts and achieved a lot in the research of cancer cachexia, so far, no effective medical intervention completely reverses cachexia, and there are no approved drug therapies [9, 10].

Skeletal muscle loss, encompassing reductions in both mass and function, is the defining clinical feature of cancer cachexia and is closely linked to functional decline and poor outcomes [2]. Pathological fat infiltration of skeletal muscle, typically reflected by accumulation of intramuscular fat (IMF; myosteatosis), accompanies or exacerbates this process [11]. Excess IMF weakens contractile force, perturbs lipid metabolism and feeds back on whole‐body metabolic control. Mechanistically, cancer cachexia features heightened proteolysis, primarily through autophagy and the UPP, together with suppressed protein synthesis; atrophy occurs when degradation outpaces synthesis [12, 13].

ActA, a key TGF‐β family ligand in the myostatin/activin pathway, is elevated in many cancers and particularly in cancer cachexia [14, 15]. As a central mediator of muscle wasting, ActA signals through two kinase receptors known as activin receptor type 2A (ACVR2A) and 2B (ACVR2B) to activate downstream Smad2/3, which in turn upregulates the E3 ubiquitin ligases Atrogin‐1 and MuRF1 and suppresses the Insulin/IGF1‐AKT–mTOR pathway. This process enhances proteolysis and accelerates muscle atrophy [16]. Although tumour‐derived Activin A is well established, the relative contribution of nontumour tissues to circulating ActA levels remains unclear.

Ligand‐trap or receptor‐level interventions targeting this pathway mitigate atrophy and improve outcomes in preclinical models but do not fully reverse cachexia. Although tumour and cancer‐associated fibroblasts are recognized sources of ActA, emerging data indicate that nontumour organs—including the kidney and heart—also contribute to ActA production in disease states. We hypothesized that ActA derived from host organs (heart and kidney) contributes significantly to circulating ActA and promotes muscle wasting and adipose tissue loss during cancer cachexia.

Although ActA acts as a detrimental regulator of skeletal muscle, it plays critical roles in early embryonic development [17], wound healing [18], neuronal proliferation [19], immune response regulation [20] and germ cell development [21]. Consequently, its complete deletion is precluded. Using tissue‐specific AAV‐mediated knockdown, we demonstrate that reducing ActA in the heart and kidney attenuates muscle atrophy, decreases intramuscular fat infiltration, improves mitochondrial integrity and mitigates white adipose loss.

## 2 Materials and Methods

### 2.1 Cell Culture

LLC cells were grown in DMEM (C11995500BT, Gibco) supplemented with 10% FBS (FSP500, ExCell) and 1% P/S solution (sv30010, HyClone). These cell lines were incubated at 37°C in a humid chamber with 5% CO2. All LLC cell lines were provided by the American Type Culture Collection (ATCC). When LLC cells reached 80%–90% confluency, they were collected by PBS (C10010500BT, Gibco). The collected LLC cell suspension is counted for viability and number using the Thermo Fisher Scientific automated cell counter, with a viability exceeding 90% (determined by trypan blue exclusion). The final cell concentration was adjusted to 1 × 10^6^cells/100ul PBS for subcutaneous implantation.

### 2.2 Mice

Six‐week‐old male C57BL/6 mice were purchased from Baineng Biological Technology Co. Ltd. (Guangzhou, China). All mice were acclimated to the environment for 1 week upon arrival. All mice were single‐housed under 12‐h light/dark cycles, at 23°C ± 2°C with 40%–60% humidity, provided with standard pellet food (M10110C1, Moldiets) and given free access to food and water in a barrier facility at the Laboratory Animal Center of Zhongke Industry Holdings Co. Ltd. Mice were then randomly assigned to four groups to ensure similar average body weights across the groups: the non–tumour‐bearing (NTB) group, the LLC cancer cachexia (LLC) group, the LLC cancer cachexia with shRNA (Inhba) AAV (sh‐Inhba) group and the LLC cancer cachexia with negative control AAV (sh‐NC) group (*N* = 6–16/group).

### 2.3 LLC Cachexia Mice Model

One week before tumour transplantation, at 7 weeks of mice age, body weight and food intake were measured daily for 7 days as baseline. LLC mice were injected with 100 μL of PBS containing 1 × 10^6^ LLC cells subcutaneously into hind limbs. The NTB mice were injected with 100 μL PBS as negative control group. The day of tumour transplantation was designated as Day 0. Mouse grip strength was measured on Day 24, and the mice were sacrificed for dissection on Day 25. For both baseline period and tumour growth period until dissection, body weight and food weight were measured and recorded daily between 4 and 7 PM. Food intake was calculated by subtracting the remaining food weight in the cage from the food weight of the previous day.

### 2.4 AAV Pilot Study

The three AAV 9 vectors carrying shRNA to interrupt the expression of *Inhba* were designed and constructed by OBiO Technology Corp. Ltd. (Shanghai, China). An additional nine 8‐week‐old male mice were divided into three weight‐matched groups of three mice each for a preliminary experiment to screen for the shRNA sequence with the highest knockdown efficiency. Briefly, 50 μL of CMV‐shRNA (Inhba)‐AAV (1 × 10^11^ vg) was injected into left renal pelvis. In the meanwhile, the right renal pelvis of the same mice was injected with control vectors (sh‐NC). Each AAV was administered to three mice through the same operation. During the entire experimental process, the mice were anaesthetized with isoflurane and maintained body temperature with a homeothermic heating blanket. After awakening spontaneously, the mice were put into their original cages. After 3 weeks when AAV became active, the mice were euthanized and kidney tissues were collected for western blot analysis to verify the knockdown efficiency by comparing the ActA expression levels of the left and right kidneys in the same mouse. Only one shRNA (Inhba) successfully reduced ActA. The sequences for shRNA (NC) were 5′‐TTCTCCGAACGTGTCACGT‐3′, while the sequences for shRNA (Inhba) were 5′‐TGGCAAGTTGCTGGATTATAG‐3′.

### 2.5 LLC Cancer Cachexia Model With hTCF21/cTNT/CMV AAV

When the mice reached 8 weeks of age, dual AAV vectors composed of hTCF21‐ and cTNT‐shRNA (Inhba) mixed at a 1:1 ratio (3 × 10^11^ vg each, in 100‐μL total volume) were injected into the sh‐Inhba group mice via the tail vein. The hTCF21 and cTNT promoters targeted cardiac fibroblasts and cardiomyocytes, respectively. On the second day, 50 μL of CMV‐shRNA (Inhba) (1 × 10^11^ vg) was injected into the bilateral renal pelvis. At the same time, same as the aforementioned procedure, the sh‐NC group mice were injected with control AAV vectors. LLC tumour cells' subcutaneous implantation was performed 3 weeks later to generate an LLC‐induced cancer cachexia mouse model. We monitored the mice's body weight and food intake daily, starting 1 week before tumour transplantation and continuing until dissection on Day 25. Additionally, we measured the grip strength on Day 24 posttransplantation.

### 2.6 Dissection

The mice were fasted for 12–16 h before dissection, with water still available. Twenty‐five days after tumour transplantation, all mice were euthanized by CO_2_ asphyxia. We obtained blood through cardiac puncture, and blood samples were placed on ice for at least 30 min, then centrifuged at 3500 rpm for 30 min at 4°C to collect serum. The serum was snap‐frozen in liquid nitrogen and stored at −80°C. The tissues of some mice (*n* = 3–5/group) were randomly selected, collected, weighed, immersed in 4% paraformaldehyde and stored at room temperature for subsequent pathological examination, while the tissues of the remaining mice were rapidly harvested, weighed, snap‐frozen in liquid nitrogen and stored at −80°C. We obtained the weights of the heart, kidneys, quadriceps, fat and tumour through dissection and weighing. The tissue weight values were all normalized to body weight (with tumour).

### 2.7 Western Blotting

Quadriceps, kidney, heart and tumour samples were lysed in ice‐cold RIPA buffer (P0013B, Beyotime) with the protease inhibitor Cocktail (B14002, Selleck) and Phosphatase Inhibitor Cocktail (B15001, Selleck). After determining the total protein concentration of the supernatant, equal amounts of proteins from different groups were resolved via 4%–20% SDS–PAGE (ET15420Gel, ACE) and then transferred onto PVDF membranes (IPVH00010, Merck Millipore). Subsequently, the membranes were blocked for 2 h at room temperature with 5% skim milk (1 706 404, BIO‐RAD) and then incubated at 4°C overnight with primary antibodies against Activin A (NBP1‐30928, Novus, 1:250), MuRF1 (ab172479‐100, Abcam, 1:1000), Atrogin‐1 (ab168372‐100, Abcam, 1:1000), CPT1B (DF3904, Affinity Biosciences, 1:1000), SCD1 (HA500119, huabio, 1:1000), β‐tubulin (S0B0238, STARTER, 1:5000), GAPDH (R380626‐100 μL, ZENBIO, 1:5000) and β‐actin (R380624‐100ul, ZENBIO, 1:5000). Then, the membranes were incubated with secondary antibodies (7074S, CST, 1:5000) at 4°C for 2 h. The target protein bands were visualized with Chemical Imaging System. The PVDF membranes were washed three times with TBST for 10 min each before proceeding to the next step. Band intensities were quantified using ImageJ software.

### 2.8 RT‐qPCR Analysis

Total RNA from mice tissue samples was extracted by an RNA Extraction Reagen (DP419, TIANGEN). Reverse transcription assay was performed in a 20 μL of reaction volume with 1 μg of total RNA by using the Hifair II first strand cDNA synthesis kit (11141ES60, YEASEN). Quantitative PCR was carried out using SYBR Green Master (E096‐01A, novoprotein) or TaqMan Fast Advanced Master Mix (4 444 554, ThermoFisher). The TATA‐box binding protein (TBP) was used as the internal reference gene for adipose tissue and β‐actin for all other tissues. Relative RNA levels were analysed with the 2^−ΔΔCT^ method. The primers used in RT‐qPCR are listed in the Supporting Information Tables S1 and S2.

### 2.9 ELISA Detection

Mouse serum levels of Activin A (ELK5382, ELK Biotechnology), BNP (ELK5227, ELK Biotechnology) and cTnT (ELK6207, ELK Biotechnology) were measured using enzyme‐linked immunosorbent assay (ELISA) kits according to the manufacturer's instructions. Each sample requires 100 μL of serum.

### 2.10 Grip Strength Test

In order to evaluate the function of mouse muscle, we used a grip strength meter (cat. no.: ZS‐ZL) to determine the maximum peak force generated by the forelimbs of the mice on the day before dissection. First, place the experimental mouse on the grip strength meter and gently pull the mouse's tail to encourage the forelimbs to grip the probe firmly. When the gradually increasing force is applied to the mouse until its forelimbs are released from the grip strength meter, record the peak reading. Three measures were made with a time interval of 10 s each time. The average value (*N*) was used.

### 2.11 Histology

Quadriceps, kidney, heart and adipose tissue were fixed, embedded and then sectioned. The slides were deparaffinized, rehydrated and stained with haematoxylin and eosin (H&E) (G1076, Servicebio), periodic acid–Schiff (PAS) (G1008, Servicebio) and Masson's trichrome stain kit (G1006, Servicebio). All procedures were carried out strictly in accordance with the standard instructions. Frozen quadriceps samples were cryosectioned at 12‐μm thickness. Muscle sections were stained with Oil Red O (ORO) (G1015, Servicebio) Staining Kit following manufacturer's instruction. Cross‐sectional areas (CSAs) of muscle tissue and adipose tissue (HE staining) were quantified by ImageJ program. Over 200 cells per section were randomly selected to measure the average CSA and fibre size distribution. The percentage of muscle fibrosis (blue collagen area/total muscle area*100%), fatty infiltration (lipid droplet area/total muscle area*100%) and glycogen content (magenta‐stained area/total muscle area*100%) were calculated using the ImageJ program [22].

### 2.12 Immunohistochemical Staining (IHC)

The slides underwent dewaxing, quenching of endogenous peroxidase activity, antigen retrieval, blocking and then were incubated with the primary antibody against Activin A (NBP1‐30928, Novus, 1:600) at 4°C overnight. After washing with PBS for three times, slides were incubated with secondary antibody for 1 h at room temperature. Immune complexes were detected with diaminobenzidine (DAB), and the sections were then dehydrated, mounted and scanned images with scanner. IHC positive staining was quantitatively analysed using ImageJ software by applying colour deconvolution to at least five fields per slide to separate DAB and haematoxylin signals and presented as average optical density (AOD) values (AOD = integrated density/Area).

### 2.13 Statistical Analysis

All data are expressed as the mean ± standard error. Student's *t*‐test was used to compare the means of two independent groups. One‐way analysis of variance (ANOVA) was performed to compare the means of multiple groups. Linear regression was used to assess the relationship between muscle weight and serum creatinine concentrations. Analysis of covariance (ANCOVA), with body weight (with tumour) as a covariate, followed by Bonferroni post hoc tests, was used to analyse differences in grip strength between groups. Statistical significance was set at *p* < 0.05.

## 3 Results

### 3.1 ActA Was Highly Expressed in Kidney and Heart of the Lewis Lung Carcinoma Cachexia Mouse Model

**FIGURE 1:**
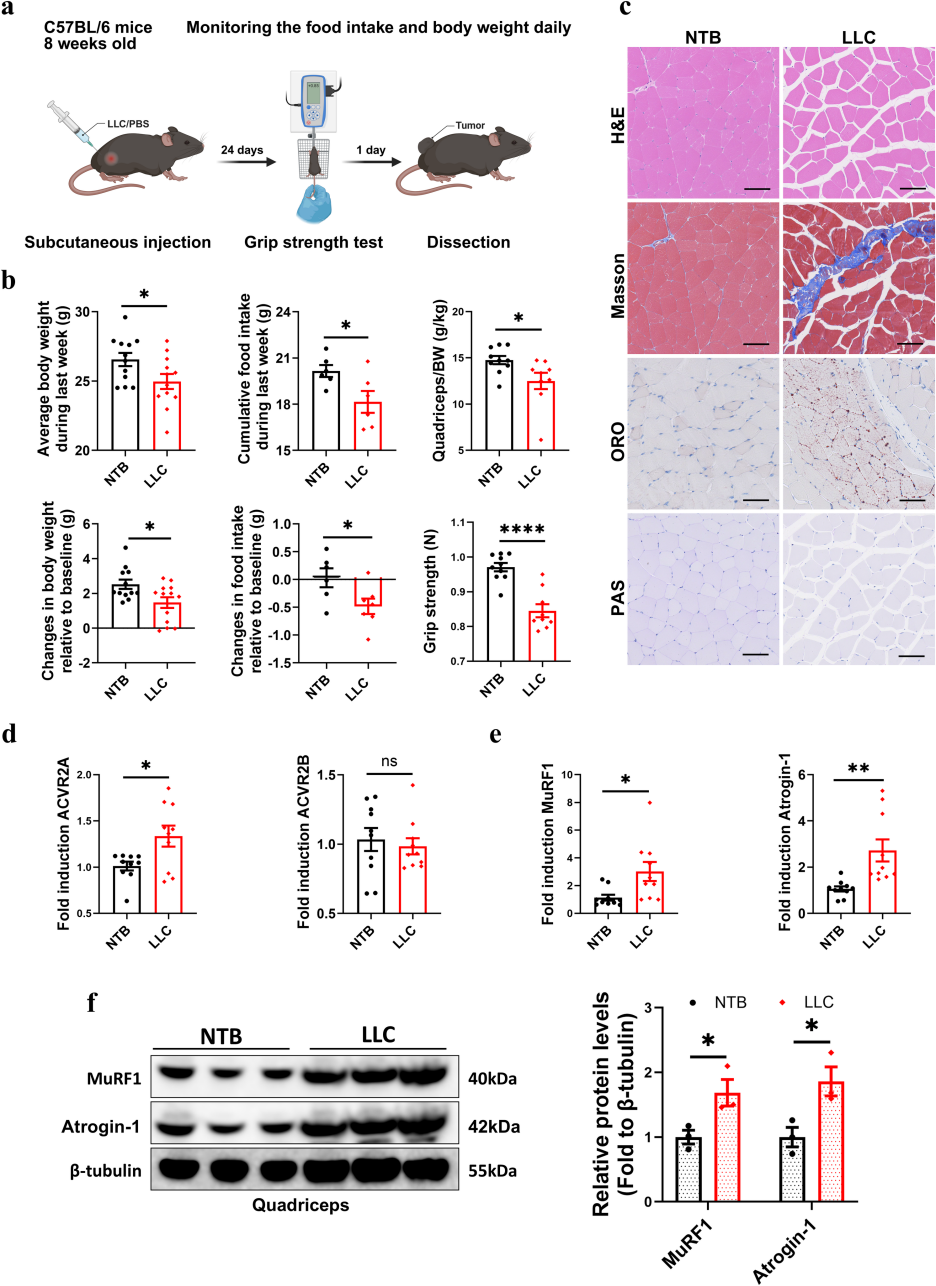
Cancer cachexia caused severe muscle loss with the upregulation of the UPP. (a) Schematic of study. (b) Average body weight during last week (g) and changes in body weight relative to baseline (g) (*n* = 12/group); cumulative food intake during last week (g) and changes in food intake relative to baseline (g) (*n* = 6–7/group); the ratio of quadriceps/body weight (g/kg) and the forelimb grip strength (*N*) (*n* = 9–10/group). (c) Representative H&E, masson, oil red O and PAS staining images of quadriceps. Scale bar = 100 μm. (d) Quantitative RT‐PCR analysis of ACVR2A and ACVR2B to β‐actin in quadriceps (*n* = 10/group). (e) Quantitative RT‐PCR analysis of MuRF1 and Atrogin‐1 to β‐actin in quadriceps (*n =* 10/group). (f) The protein levels of MuRF1 and Atrogin‐1 in quadriceps were evaluated by western blot and representative images were shown (*n* = 3/group).

LLC (1 × 10^6^ cells/100ul PBS) was injected subcutaneously into the hind limbs of mice with daily measuring body weight and food intake, and the mice were euthanized and dissected after 25 days (Figure 1a). As shown in Figure 1b, the body weight and food intake of LLC mice were significantly decreased compared with NTB mice (*t*‐test, *p* < 0.05). LLC mice exhibited a significantly lower muscle‐to‐body‐weight ratio (*t*‐test, *p* < 0.05) and grip strength compared to NTB group. ANCOVA with body weight (with tumour) as a covariate showed that there were significances between different groups for grip strength with no differences due to body weight or the groups*body weight interaction (ANCOVA, *F*_(1,17)_ = 2.045, *p* > 0.05 for groups*body weight; *F*_(1,17)_ = 0.962, *p* > 0.05 for body weight; *F*_(1,17)_ = 30.285, *p* < 0.0001 for groups). Next, we compared muscle histopathology between the two groups and found the LLC mice exhibited smaller myofiber CSA, more severe muscle fibrosis, greater intramuscular fat infiltration and lower glycogen content than NTB mice (Figures 1c and S1a–d), which suggests significant muscle damage in cancer cachexia. The UPP was markedly upregulated in muscle of LLC mice. The LLC mice had an increasing ACVR2A mRNA level without changes in ACVR2B (*t*‐test, *p* < 0.05 for ACVR2A; *p* > 0.05 for ACVR2B) (Figure 1d). MuRF1 and Atrogin‐1 expressions were markedly increased at both the mRNA and protein levels (Figure 1e,f), consistent with enhanced proteolysis and accelerated muscle atrophy. Taken together, the hallmark features of cachexia, anorexia, rapid weight loss and muscle atrophy were reproduced in our LLC cachexia mice model.

**FIGURE 2:**
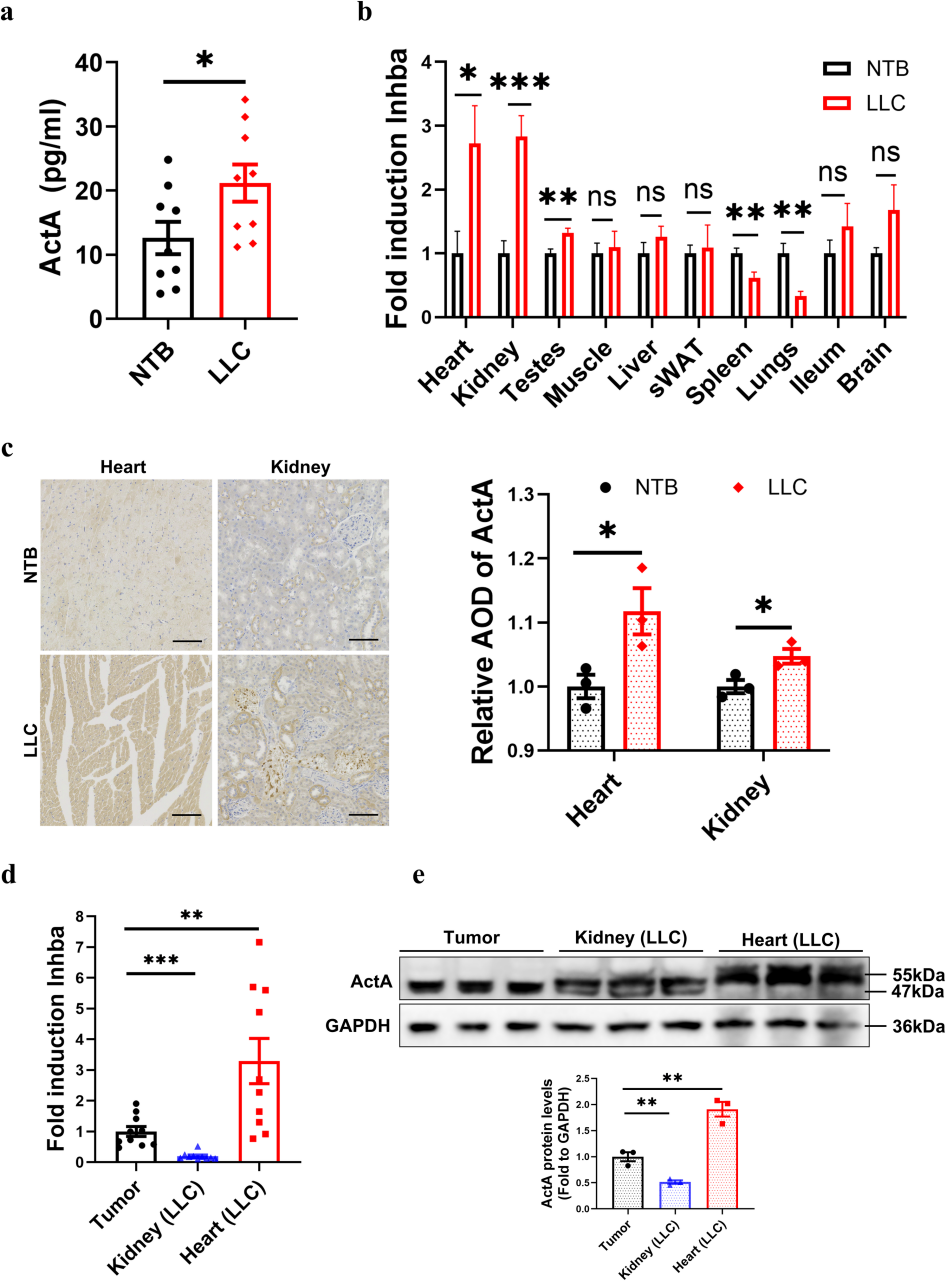
ActA was highly expressed in kidney and heart of LLC mice. (a) ActA blood level (*n =* 9/group). (b) Quantitative RT‐PCR analysis of *Inhba* to β‐actin in different organs (*n =* 9–10/group). (c) Representative IHC staining images of ActA in heart and kidney and the quantified analysis by ImageJ (*n =* 3/group). Scale bar = 100 μm. (d) Quantitative RT‐PCR analysis of Inhba to β‐actin in tumour, heart and kidney of LLC mice (*n* = 10/group). (e) The protein levels of ActA in tumour, kidney and heart were evaluated by western blot and representative images were shown (tumour: 47 kDa, kidney: 47 kDa, Heart: 55 kDa; *n* = 3/group).

Tumours produced high levels of ActA, which contribute to skeletal muscle atrophy in cancer cachexia [14]. The serum ActA level was significantly increased in LLC mice compared to NTB mice (*t*‐test, *p* < 0.05) (Figure 2a). More and more evidence indicates that nontumour organs, including the kidney (e.g., in chronic kidney disease) [23] and the heart (e.g., in heart failure) [24], also produce ActA. To explore whether other tissues contribute to ActA in cancer cachexia, we examined *Inhba,* the gene encoding ActA, across multiple tissues in LLC mice (Figure 2b). Interestingly, the mRNA levels of *Inhba* in the heart (*p* < 0.05) and kidney (*p* < 0.001) in LLC mice were elevated nearly threefold than NTB mice. We further applied IHC to detect the distribution and content of ActA protein and confirmed tha ActA protein levels both in the heart and kidney were significantly increased in LLC mice compared to NTB (Figure 2c). Across tissues, *Inhba* was significantly elevated in tumour, heart and kidney in LLC mice, with heart *Inhba* mRNA and protein levels exceeding tumour (both *p* < 0.01) (Figure 2d,e). These findings prompted us to test whether ActA from heart‐ and kidney‐sources contribute functionally to cachexia phenotypes in vivo.

### 3.2 The Mass and Function of Heart and Kidney Were Seriously Impaired in Cancer Cachexia Mice

**FIGURE 3:**
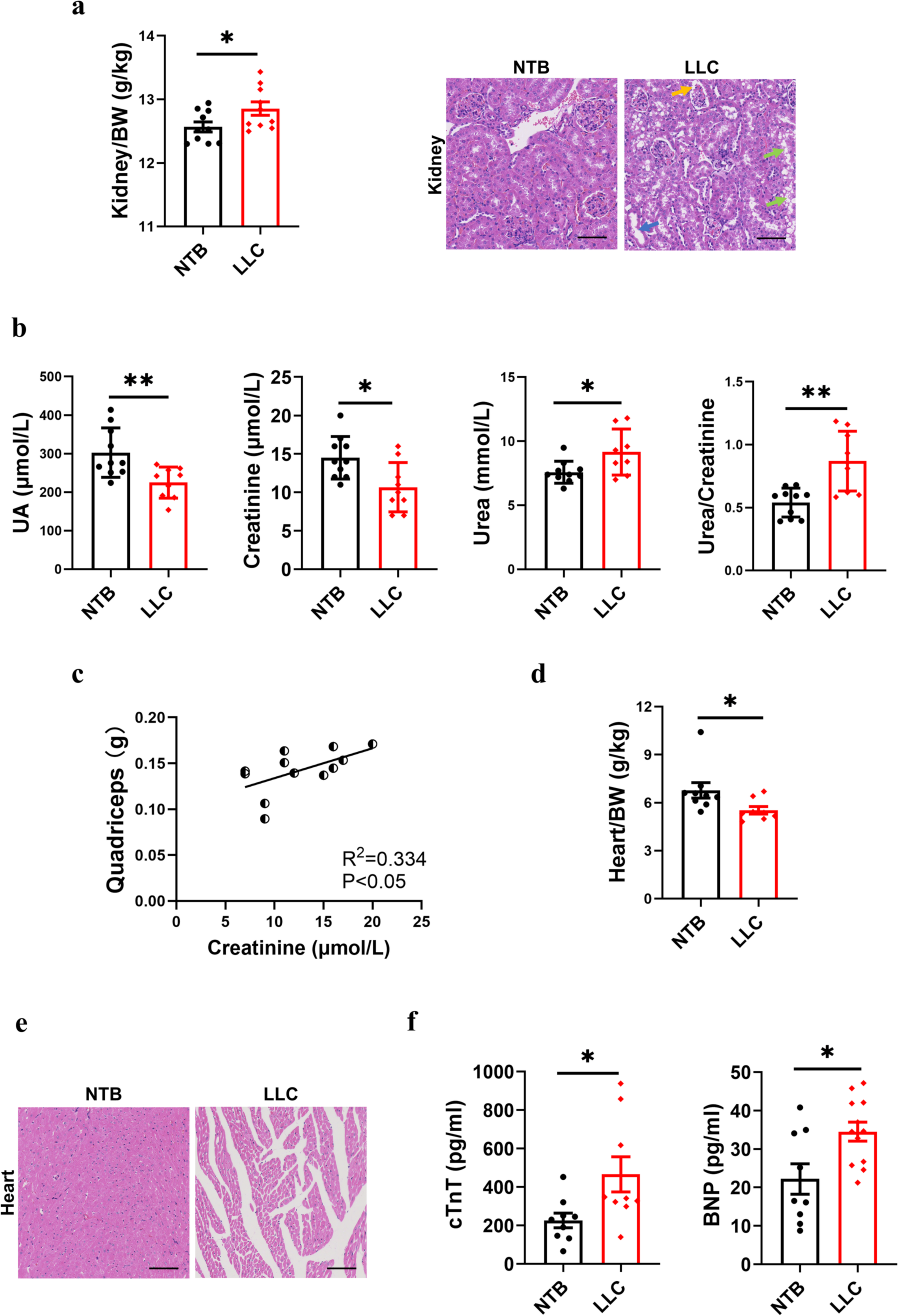
Cancer cachexia caused severe damage to heart and kidney. (a) The ratio of kidney/body weight (g/kg) (*n* = 10/group) and representative H&E staining images of kidney. Scale bar = 100 μm. (b) UA, urea and creatinine blood levels and the ratio of urea/creatinine (mmol/μmol) in blood (*n* = 8–10/group). (c) Regression analysis between quadriceps weight and creatinine blood level (*n* = 11/group). (d) The ratio of heart/body weight (g/kg) (*n* = 8–9/group). (e) Representative H&E staining images of heart. Scale bar = 100 μm. (f) cTnT and BNP blood levels (*n* = 9–12/group).

We first determined whether the heart and kidney functions have been compromised in cancer cachexia. As shown in Figure 3a, kidney‐to‐body‐weight ratio significantly increased in LLC mice compared to NTB mice (*t*‐test, *p* < 0.05), and the H&E staining of kidney defined the pathological changes including a dilation of renal tubular (blue arrow), foamification of renal tubular (green arrows) and renal capsules (orange arrow). In LLC mice, plasma uric acid (UA) and creatinine concentrations dramatically decreased (*t*‐test, *p* < 0.01 for UA; *p* < 0.05 for creatinine), while serum urea levels markedly increased (*t*‐test, *p* < 0.05) (Figure 3b). This resulted in a substantial increase in the urea‐to‐creatinine ratio, which is primarily attributable to impaired renal function and reduced muscle mass. Creatinine is primarily produced by muscle metabolism. The LLC mice with muscle loss and less muscle mass have lower creatinine levels (linear regression, *p* < 0.05) (Figure 3c).

Similarly, we examined the abnormalities in the histopathological structure and biochemical indicators of the heart. Heart‐to‐body‐weight ratio declined significantly in LLC mice (*t*‐test, *p* < 0.05) (Figure 3d) and the pathological finding explained the decrease in weight (Figure 3e). The reduction in the size and number of cardiomyocytes, along with the appearance of thin and sparse myocardial fibres, accompanied an increase in the interstitial space between the myocardial fibres, which were the typical characteristics of myocardial atrophy. A notable rise in serum Cardiac Troponin T (cTnT) and B‐type Natriuretic Peptide (BNP) of LLC mice had been detected (*t*‐test, *p* < 0.05), indicating that the heart had likely suffered ischemic injury and heart failure (Figure 3f). In short, the kidney and heart had suffered severe damage during cancer cachexia process.

### 3.3 Knocking Down the Expression of ActA in Heart and Kidney Ameliorated Body Weight Loss and Anorexia of LLC Mice

**FIGURE 4:**
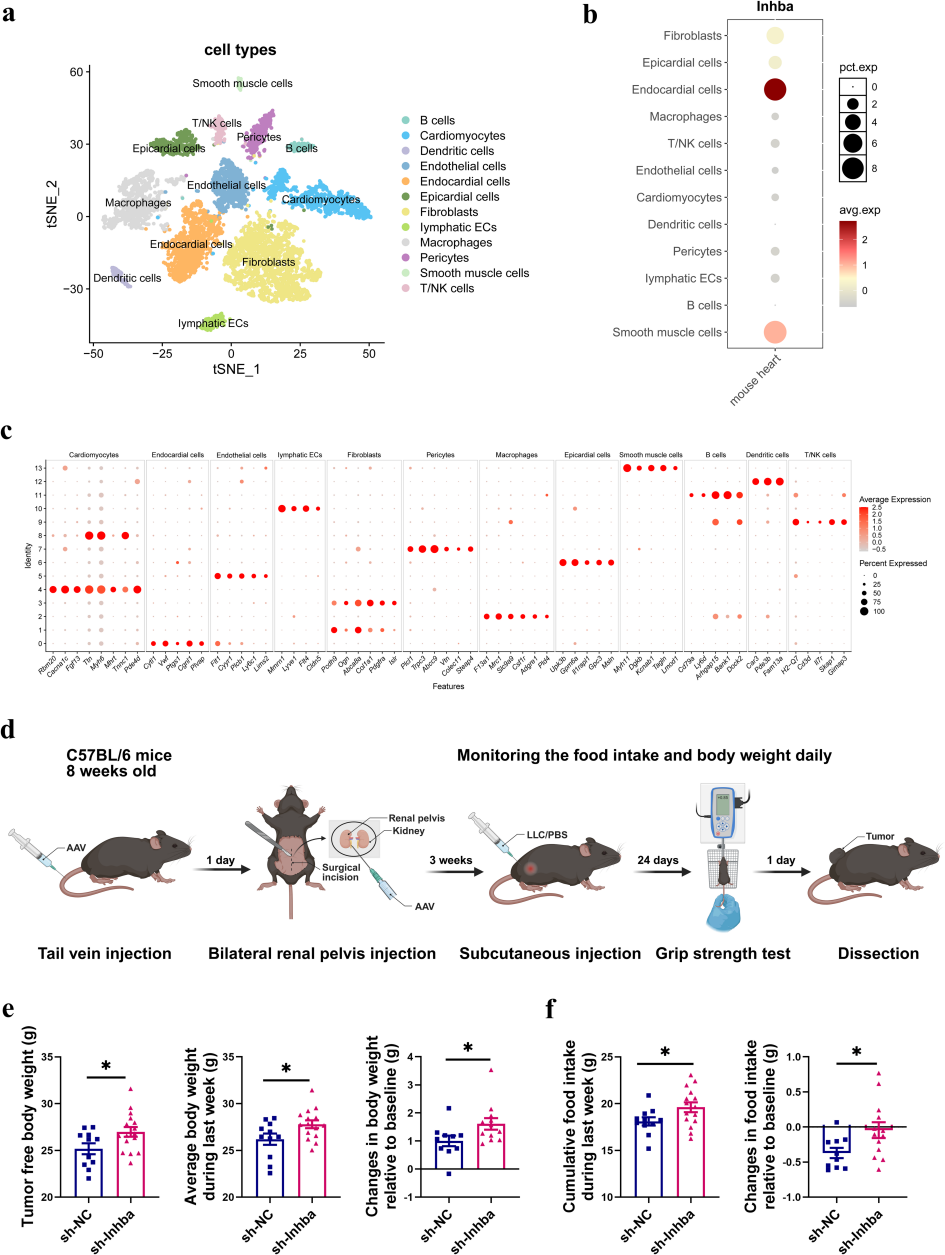
Knocking down the expression of ActA in heart and kidney ameliorated bodyweight loss and anorexia of LLC mice. (a) t‐distributed stochastic neighbour embedding (t‐SNE) projection of cardiac cells from mouse heart (ECs: endothelial cells). (b) Dot plot showing the expression of *Inhba* in the mouse heart. (c) The expression levels of top 5 distinct marker genes in each cell type. (d) Schematic of study. (e) The body weight without tumour, the average body weight during last week and the changes in body weight relative to baseline (*n* = 10–16/group). (f) Cumulative food intake during last week and changes in food intake relative to baseline (*n* = 10–15/group).

To identify the cell population expressing *Inhba* in the murine heart, we have queried the Gene Expression Omnibus (GEO) and found single‐nucleus transcriptome sequencing (snRNA‐seq) data of mouse hearts (GSM8691126). Twelve different types of cells in the mouse heart were identified by unsupervised clustering and expression of lineage‐specific markers [25] following batch correction (Figure 4a,c). We found that *Inhba* was predominantly expressed in fibroblasts, epicardial cells, endocardial cells and smooth muscle cells (Figure 4b). However, other cell types in the heart exhibited minimal expression of *Inhba*. Previous research has established that *Inhba* expression is exclusive to cells within the juxtaglomerular apparatus [26]. Consistent with the results from mouse tissues, publicly available single‐cell transcriptome sequencing (scRNA‐seq data: GSE131882 & GSE134355) suggests that the human heart and kidney also can express *INHBA* (Figure S2a–f).

To investigate the role of ActA generated by host organs (heart and kidney) in LLC mice, we first performed a pilot study to identify AAV constructs with the greatest *Inhba* knockdown efficiency (Figure S3a,b). We then delivered the selected tissue‐restricted AAVs to the host organs and performed implantation of LLC cells subcutaneously 3 weeks later (Figure 4d). Twenty‐five days after implantation, the mice were euthanized and dissected. We imaged fluorescence in the host organs (Figure S3c) and other tissues (Figure S4) and confirmed efficient organ‐specific AAV transduction. *Inhba* knockdown in host organs did not alter the protein levels in tumour or tumour weight (*t*‐test, both *p* > 0.05) (Figure S3d), whereas ActA protein levels were significantly reduced in the targeted host organs (Figure S3e).

Compared to the sh‐NC group, the sh‐*Inhba* group showed significant increases in tumour free body weight, average final week body weight and the body weight changes relative to baseline (*t*‐test, all *p* < 0.05) (Figure 4e). The sh‐*Inhba* group significantly increased cumulative food intake over the last 7 days and the changes in food intake relative to baseline (*t*‐test, both *p* < 0.05) (Figure 4f). Taken together, *Inhba* knockdown in host organs improved body weight and food intake in cachectic mice.

### 3.4 Downregulation ActA in Heart and Kidney Attenuated the Muscle Atrophy and Intramuscular Fat Infiltration in LLC Mice

**FIGURE 5:**
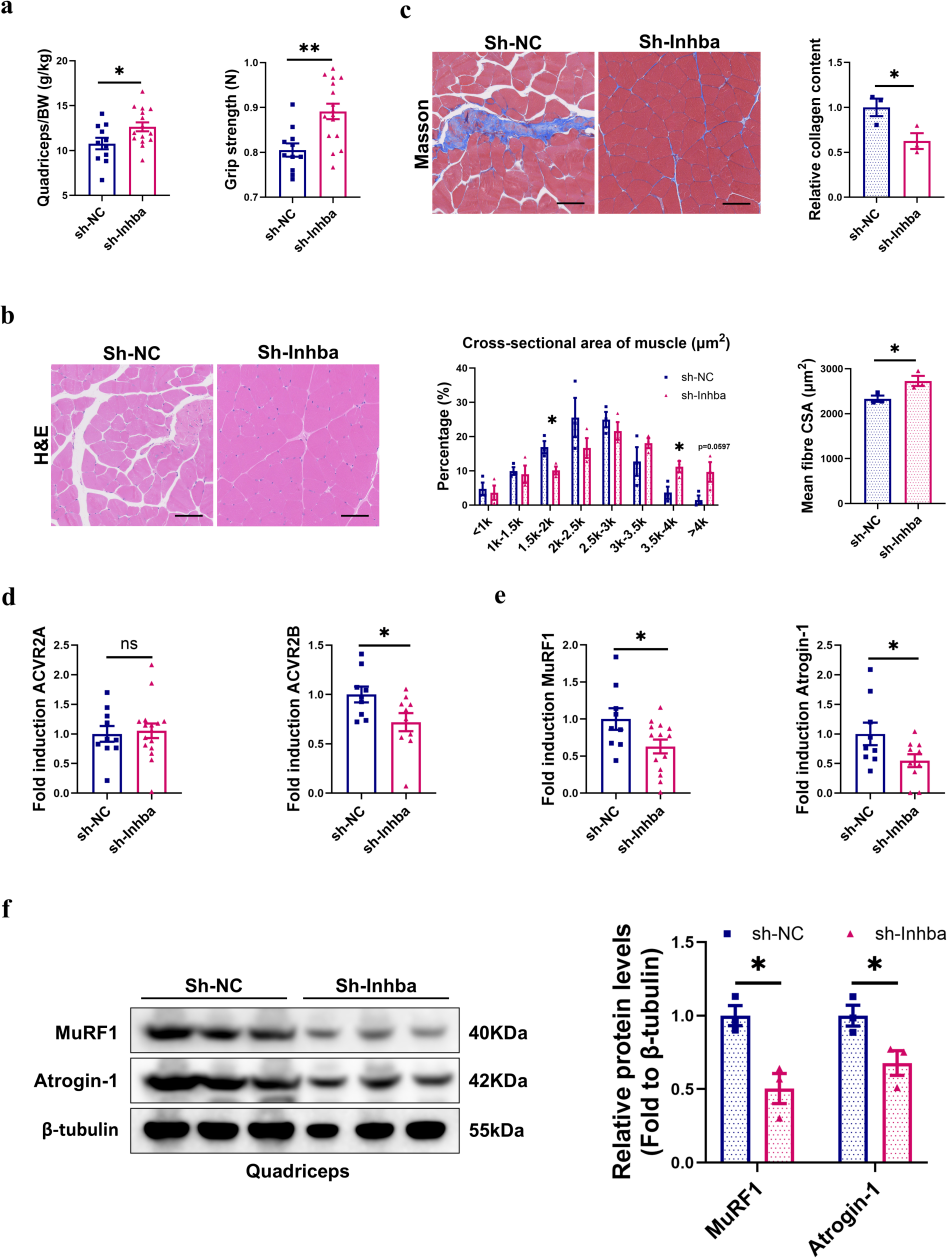
Downregulation ActA in heart and kidney attenuated the muscle atrophy in LLC mice. (a) The ratio of quadriceps/body weight (g/kg) and the forelimb grip strength (*N*) (*n* = 11–16/group). (b) Representative H&E staining images of quadriceps and the quantified analysis by ImageJ (*n* = 3/group). Scale bar = 100 μm. (c) Representative masson staining images of quadriceps and the quantified analysis by ImageJ (*n* = 3/group). Scale bar = 100 μm. (d) Quantitative RT‐PCR analysis of ACVR2A and ACVR2B to β‐actin in quadriceps (*n* = 9–16/group). (e) Quantitative RT‐PCR analysis of MuRF1 and Atrogin‐1 to β‐actin in quadriceps (*n* = 9–14/group). (f) The protein levels of MuRF1 and Atrogin‐1 in quadriceps were evaluated by western blot and representative images were shown (*n* = 3/group).

To explore the impact of host organs on muscle mass and function, we isolated the quadriceps from the sh‐NC and sh‐*Inhba* group. The sh‐*Inhba* group mice with lower ActA in host organs significantly increase muscle mass (*t*‐test, *p* < 0.05) and improve the forelimb grip strength (Figure 5a). ANCOVA with body weight (with tumour) as a covariate showed that there were significances between different groups for grip strength with no differences due to body weight or the groups*body weight interaction (ANCOVA, *F*_(1,25)_ = 0.268, *p* > 0.05 for groups*body weight; *F*_(1,25)_ = 3.415, *p* > 0.05 for body weight; *F*_(1,25)_ = 9.579, *p* < 0.01 for groups). H&E staining revealed that the sh‐*Inhba* mice showed greater mean fibres CSA than the sh‐NC group, especially with the CSA differences of 1.5–2 k and 3.5–4 k between the two groups being particularly significant (Figure 5b). The sh‐*Inhba* mice showed a lower collagen content in muscle than sh‐NC mice (Figure 5c), indicating attenuated muscle fibrosis with lower ActA in heart and kidney.

There was no significant difference in the mRNA levels of ACVR2A. However, the sh‐*Inhba* group showed a significantly downregulated mRNA levels of ACVR2B compared with the sh‐NC group (*t*‐test, *p* > 0.05 for ACVR2A, *p* < 0.05 for ACVR2B) (Figure 5d). The sh‐*Inhba* group showed significant declines in the mRNA and protein levels of MuRF1 and Atrogin‐1 compared with the sh‐NC group (Figure 5e,f). Overall, these data provided profound evidence that lowering the ActA in heart and kidney of LLC mice ameliorated muscle wasting by diminishing the UPP.

**FIGURE 6:**
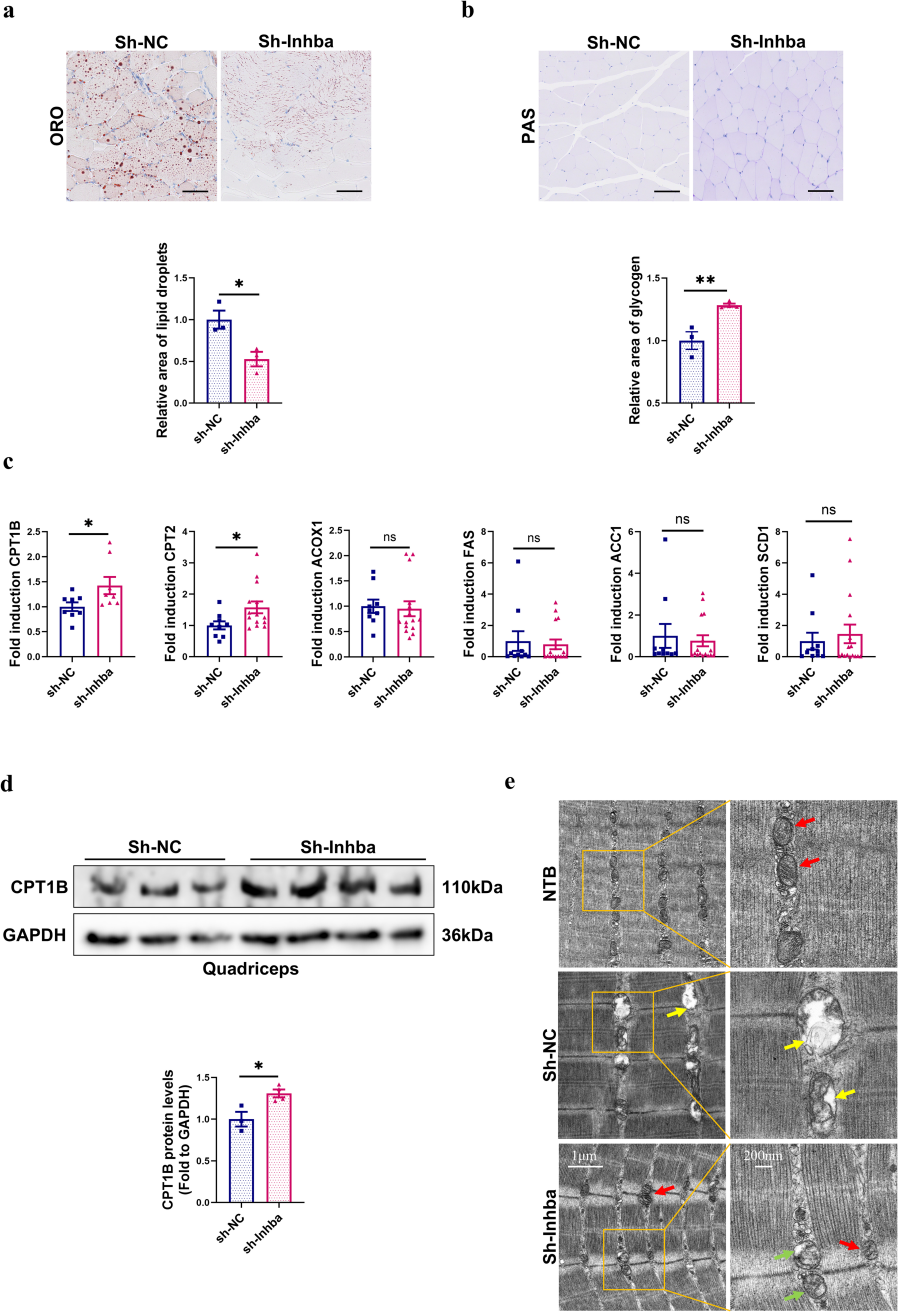
Downregulation ActA in heart and kidney attenuated intramuscular fat infiltration in LLC mice. (a) Representative ORO staining images of quadriceps and the quantified analysis by ImageJ (*n* = 3/group). Scale bar = 100 μm. (b) Representative PAS staining images of quadriceps and the quantified analysis by ImageJ (*n* = 3/group). Scale bar = 100 μm. (c) Quantitative RT‐PCR analysis of CPT1B, CPT2, ACOX1, FAS, ACC1 and SCD1 to β‐actin in quadriceps (*n* = 8–16/group). (d) The protein levels of CPT1B in quadriceps were evaluated by western blot and representative images were shown (*n* = 3–5/group). (e) Representative electron micrographs of quadriceps.

Decreased lipid droplets and increased glycogen deposition were observed in sh‐*Inhba* mice (Figure 6a,b), indicating an improvement of myosteatosis and energy metabolism in skeletal muscle. We measured the mRNA expression of lipid metabolism genes in skeletal muscle: fatty acid oxidation (FAO) (CPT1B, CPT2, acyl‐CoA oxidase 1 (ACOX1)) and lipogenesis (fatty acid synthase (FAS), acetyl‐CoA carboxylase 1 (ACC1), stearoyl‐CoA desaturase 1 (SCD1)) (Figure 6c). The mRNA levels of CPT1B and CPT2 were significantly higher in sh‐*Inhba* mice than in sh‐NC mice (*t*‐test, both *p* < 0.05), whereas ACOX1 showed no change in both groups (*t*‐test, *p* > 0.05). The lipogenic genes FAS*,* ACC1, and SCD1 showed no significant difference between groups (*t*‐test, all *p* > 0.05). The protein level of CPT1B was also significantly increased (Figure 6d).

CPT1B is a rate‐limiting enzyme for mitochondrial fatty‐acid β‐oxidation, which localizes to the mitochondrial outer membrane, and CPT2 is another important enzyme located in the mitochondrial inner membrane that participates in FAO [27]; we examined mitochondrial ultrastructure by transmission electron microscopy (TEM) (Figure 6e). In NTB mice, mitochondria displayed normal morphology with intact outer membranes (red arrows). The mitochondria in the sh‐NC group exhibited swollen mitochondria with disrupted membranes and disorganized cristae (yellow arrows). In sh‐*Inhba* mice, mitochondrial morphology was markedly improved, with largely preserved membrane integrity, reduced swelling and the presence of morphologically normal mitochondria (green and red arrows).

Together, these findings indicate that suppression of host‐organ–derived ActA is associated with partial restoration of mitochondrial fatty‐acid oxidation‐related markers (including CPT1B), improvement of mitochondrial architecture and attenuation of myosteatosis and muscle atrophy in LLC‐induced cancer cachexia, relative to LLC controls.

### 3.5 Blocking ActA in Heart and Kidney Mitigated White Adipose Tissue Loss in LLC Mice

**FIGURE 7:**
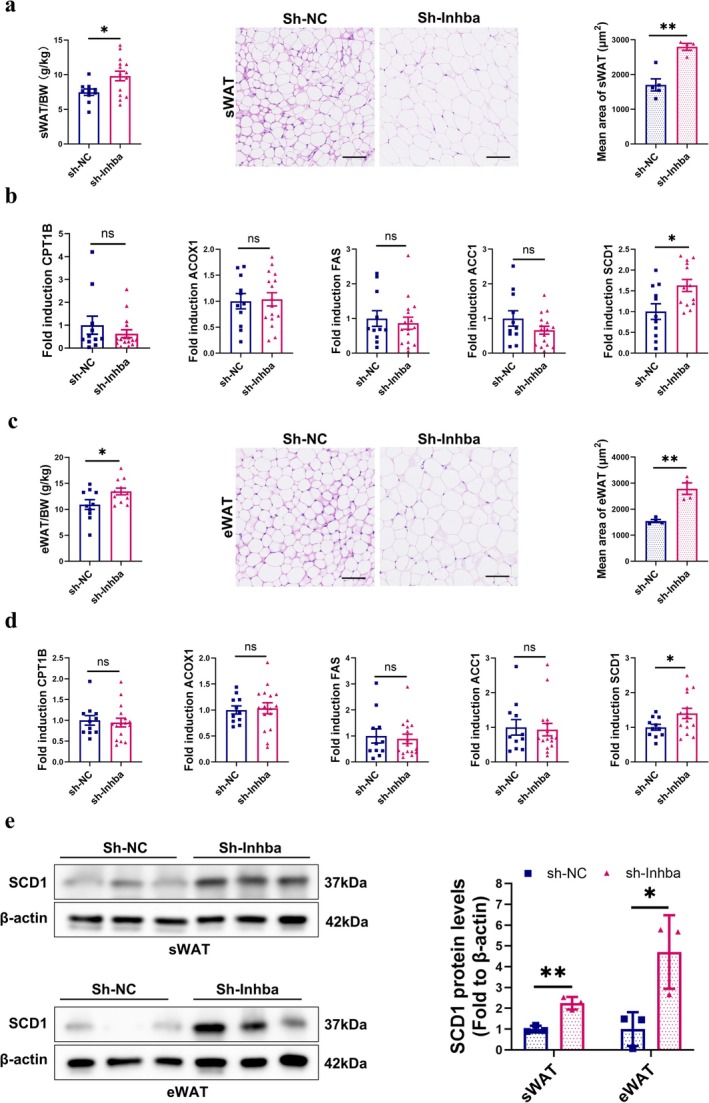
Blocking ActA in heart and kidney mitigated white adipose tissue loss in LLC mice. (a) The ratio of sWAT/body weight (g/kg) (*n* = 10–15/group); representative H&E staining images of sWAT and the quantified analysis by ImageJ (*n* = 4–5/group). Scale bar = 100 μm. (b) Quantitative RT‐PCR analysis of CPT1B, ACOX1, FAS, ACC1 and SCD1 to TBP in sWAT (*n* = 11–16/group). (c) The ratio of eWAT/body weight (g/kg) (*n* = 10–12/group); representative HE staining images of eWAT and the quantified analysis by ImageJ (*n* = 4/per group). Scale bar = 100 μm. (d) Quantitative RT‐PCR analysis of CPT1B, ACOX1, FAS, ACC1 and SCD1 to TBP in eWAT (*n* = 11–16/group). The protein levels of SCD1 in WAT were evaluated by western blot and representative images were shown (*n* = 3/group).

Some studies had suggested that increasing circulating levels of Act A were correlated with atrophy of white‐adipose‐tissue [28, 29]. We next encompassed subcutaneous (sWAT) and epididymal white adipose tissue (eWAT) into the scope of our study and evaluated whether organ‐targeted ActA knockdown would also alleviate WAT loss. We found a significant increase in fat mass (*t*‐test, *p* < 0.05) and adipocytes CSA (*t*‐test, *p* < 0.01) (Figure **7** **a** and **c**). The β‐oxidation genes (CPT1B, ACOX1) were unchanged, whereas the lipogenic enzyme SCD1 was selectively upregulated, with no differences in FAS or ACC1 in both sWAT and eWAT (Figure 7b,d). SCD1 protein level was also significantly elevated (Figure 7e). These results suggested that organ‐targeted ActA knockdown might potentially ameliorate WAT loss, possibly through elevating the expression of SCD1.

## 4 Discussion

Cancer cachexia is a complex metabolic syndrome characterized by progressive skeletal muscle and adipose tissue loss, and its underlying mechanisms remain incompletely understood. Consequently, effective therapeutic options are limited and clinical outcomes remain unsatisfactory [30]. Identifying systemic drivers beyond the tumour itself is therefore critical for improving cachexia management.

**FIGURE 8:**
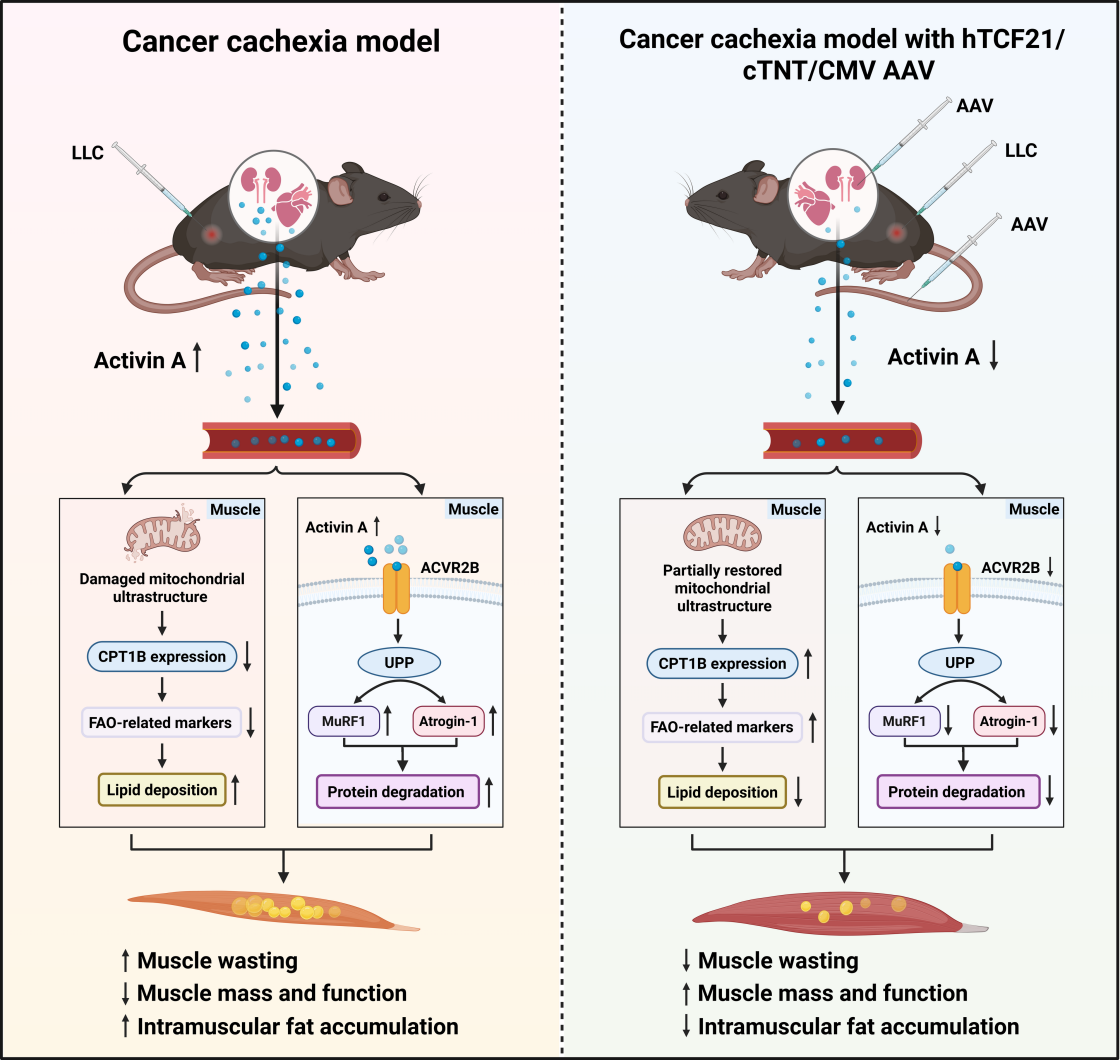
Schematic overview of the process that cancer cachexia mediated muscle atrophy and intramuscular fat infiltration through ActA produced by the heart and kidney.

In this study, we identify a pathogenic contribution of host‐organ‐derived ActA, particularly from the heart and kidney, in an LLC‐induced cachexia model. By generating an organ‐wide ActA expression atlas, we show that ActA is markedly upregulated not only in tumours but also in host organs, with especially prominent increases in the heart and kidney. Elevated ActA from these organs was associated with body weight loss, food intake reduction, skeletal muscle atrophy and WAT wasting. Importantly, organ‐targeted knockdown of ActA in the heart and kidney partially alleviated muscle loss and functional decline, reduced intramuscular lipid accumulation and mitigated WAT loss without altering tumour burden, indicating an independent contribution of host–organ‐derived ActA to cachexia severity. The possible mechanism of organ‐targeted inhibition to ActA in ameliorating cancer cachexia was illustrated in Figure 8.

Although cachexia is classically defined by skeletal muscle wasting, nonskeletal muscle organs have received comparatively little attention [31]. Here, we observed pronounced pathological changes in the heart and kidney of LLC mice, reflected by altered organ mass, abnormal histopathology and dysregulated biochemical indicators, together with robust upregulation of ActA. These findings are consistent with chronic kidney disease and cardiometabolic models, in which elevated ActA production from the kidney and heart contributes to increased circulating ActA levels [23]. Likewise, selective upregulation of *Inhba* in the kidney has been shown to be sufficient to induce skeletal muscle atrophy in nephropathy models [26]. Collectively, these data support the concept that heart‐ and kidney‐derived ActA represent important nontumour sources that amplify systemic catabolic signalling.

Previous studies have established that many tumour types secrete high levels of ActA, which promotes skeletal muscle atrophy through activation of catabolic pathways [32, 33, 34, 35]. Our findings extend this paradigm by demonstrating that host organs also contribute to circulating ActA and engage similar downstream signalling in muscle. Notably, whereas tumour‐targeted ActA inhibition improves survival without affecting food intake [34], selective attenuation of host‐organ‐derived ActA in our model increased both body weight and food intake while improving muscle and adipose phenotypes, suggesting partially distinct physiological roles for tumour‐ versus host‐derived ActA.

At muscle level, cachexia is characterized by excessive protein degradation, often mediated by the UPP [36]. In the present study, organ‐targeted ActA knockdown was associated with reduced expression of UPP‐related markers, including MuRF1 and Atrogin‐1, together with attenuation of muscle atrophy. Importantly, we interpret these changes as a partial normalization of excessive proteolysis under cachectic conditions rather than a complete suppression of pathological protein turnover. Because both sh‐NC and sh‐*Inhba* mice remained tumour‐bearing, these effects reflect modulation of disease severity rather than full reversal of cachexia.

In addition to muscle mass preservation, attenuation of host‐derived ActA was associated with reduced intramuscular fat accumulation. This phenotype coincided with increased CPT1B expression at both the mRNA and protein levels, upregulation of CPT2 mRNA and partial improvement of mitochondrial ultrastructure. As CPT1B and CPT2 are key components of the fatty acid β‐oxidation pathway, these findings are consistent with improved mitochondrial integrity and fatty acid oxidation‐related capacity. However, in the absence of direct measurements of mitochondrial respiration or β‐oxidation flux, these observations should be interpreted as supportive structural and molecular evidence rather than definitive functional enhancement.

Mitochondrial dysfunction and ectopic lipid deposition (myosteatosis) contribute to metabolic dysregulation, inflammation, and insulin resistance, thereby exacerbating muscle wasting [37], and are associated with increased cardiovascular risk [38]. A substantial increase in glycogen deposition may signify enhanced systemic insulin sensitivity and an improved capacity of muscle to take up and utilize glucose. The reduction in myosteatosis observed following organ‐targeted ActA attenuation may therefore indirectly contribute to the improvements in muscle mass and function by ameliorating metabolic status.

In adipose tissue, attenuation of heart‐ and kidney‐derived ActA was associated with reduced WAT loss and increased expression of SCD1 at both the mRNA and protein levels. Given that SCD1 deficiency leads to adipocyte death and decreases adipocyte volume and number [39], these findings suggest that host–organ‐derived ActA may influence adipose tissue maintenance, potentially through modulation of lipogenic programmes.

Several limitations should be acknowledged. First, metabolic inferences are based largely on transcriptional, protein expression and ultrastructural data rather than direct functional assays. Second, some comparisons were performed within tumour‐bearing groups, limiting conclusions regarding complete normalization to a NTB state. Finally, although localized AAV delivery was used, low‐level off‐target viral distribution cannot be entirely excluded.

In summary, our findings support a model in which heart‐ and kidney‐derived ActA act as important systemic contributors to cancer cachexia, exacerbating muscle and adipose tissue wasting in the presence of a tumour. Targeting host organ‐derived ActA does not eliminate cachexia but may slow disease progression and improve metabolic and functional outcomes. Future studies incorporating direct metabolic flux measurements, long‐term outcome analyses and clinical validation will be essential to define the translational potential of this strategy.

## 5 Conclusion

Our results suggest that downregulation of Activin A in the kidney and heart attenuates intramuscular adipogenesis, alleviates skeletal muscle atrophy, improves muscle mass and function and restores adipose mass in cancer cachexia mice. These data identify heart‐ and kidney‐derived Activin A as a key driver of wasting and support organ‐ and tumour‐targeted inhibition as a more precise and potentially safer approach than systemic blockade.

## Ethics Statement

The authors of this manuscript certify that they comply with the ethical guidelines for authorship and publishing in the *Journal of Cachexia, Sarcopenia and Muscle* [40]. All animal experiments were performed according to the protocols reviewed and approved by the Experimental Animal Welfare and Ethics Committee of Zhongke Industry Holdings Co. Ltd. (no. 202400153). The manuscript does not contain any clinical studies or patient data.

## Conflicts of Interest

The authors declare no conflicts of interest.

## Supporting information


**Figure S1:** The pathological results of quadriceps in NTB and LLC mice were quantitatively analysed using ImageJ. The fibre size distribution and the average CSA (*n* = 3/group). The relative collagen content (*n* = 3/group). The relative area of lipid droplets (*n* = 3/group). The relative area of glycogen (*n* = 3/group).

**Figure S2:** jcsm70237‐sup‐0002‐Supplementary_Figure2.TIF. *INHBA* was widely expressed in normal human kidney and heart tissues. (a) t‐SNE projection of cells from human heart demonstrating 6 cell types. (b) t‐SNE plot showing the average expression level of *INHBA* in human heart cell types. (c) Dot plot showing the expression of *INHBA* in the human heart. (d) t‐SNE projection of cells from human kidney demonstrating 11 cell types. (e) t‐SNE plot showing the average expression level of *INHBA* in human kidney cell types. (f) Dot plot showing the expression of *INHBA* in the human kidney; PCT: proximal convoluted tubule; CFH: complement factor H; LOH: loop of Henle; DCT: distal convoluted tubule; CT: connecting tubule; CD: collecting duct; PC: principal cell; IC: intercalated cell; PODO: podocyte; ENDO: endothelium; MES: mesangial cell; LEUK: leukocyte.

**Figure S3:** Knocking down the expression of ActA in heart and kidney by injecting tissue‐specific AAV. (a) Schematic of study. (b) The protein levels of ActA in kidney were evaluated by western blot after AAV administration with the greatest ActA knockdown efficiency (*n* = 3/group). (c) The fluorescence images of heart and kidney. (d) The protein levels of ActA in tumour were evaluated by western blot and representative images were shown (*n* = 3/group); the tumours weight (g) (*n* = 10–16/group). (e) Representative IHC staining images of ActA in heart and kidney. Scale bar = 100 μm.

**Figure S4:** The fluorescence images of liver, quadriceps, tumour and perirenal fat.

**Table S1:** Oligonucleotides.
**Table S2:** TaqMan primer pairs.
