# Supplementary figures and images for: Downregulation of Organ‐Derived Activin A Attenuates Muscle Atrophy and Intramuscular Fat Infiltration in Cancer Cachexia Mice
Source: J Cachexia Sarcopenia Muscle. 2026 Mar 11;17(2):e70237. doi: 10.1002/jcsm.70237 (PMC12976578; doi:10.1002/jcsm.70237)

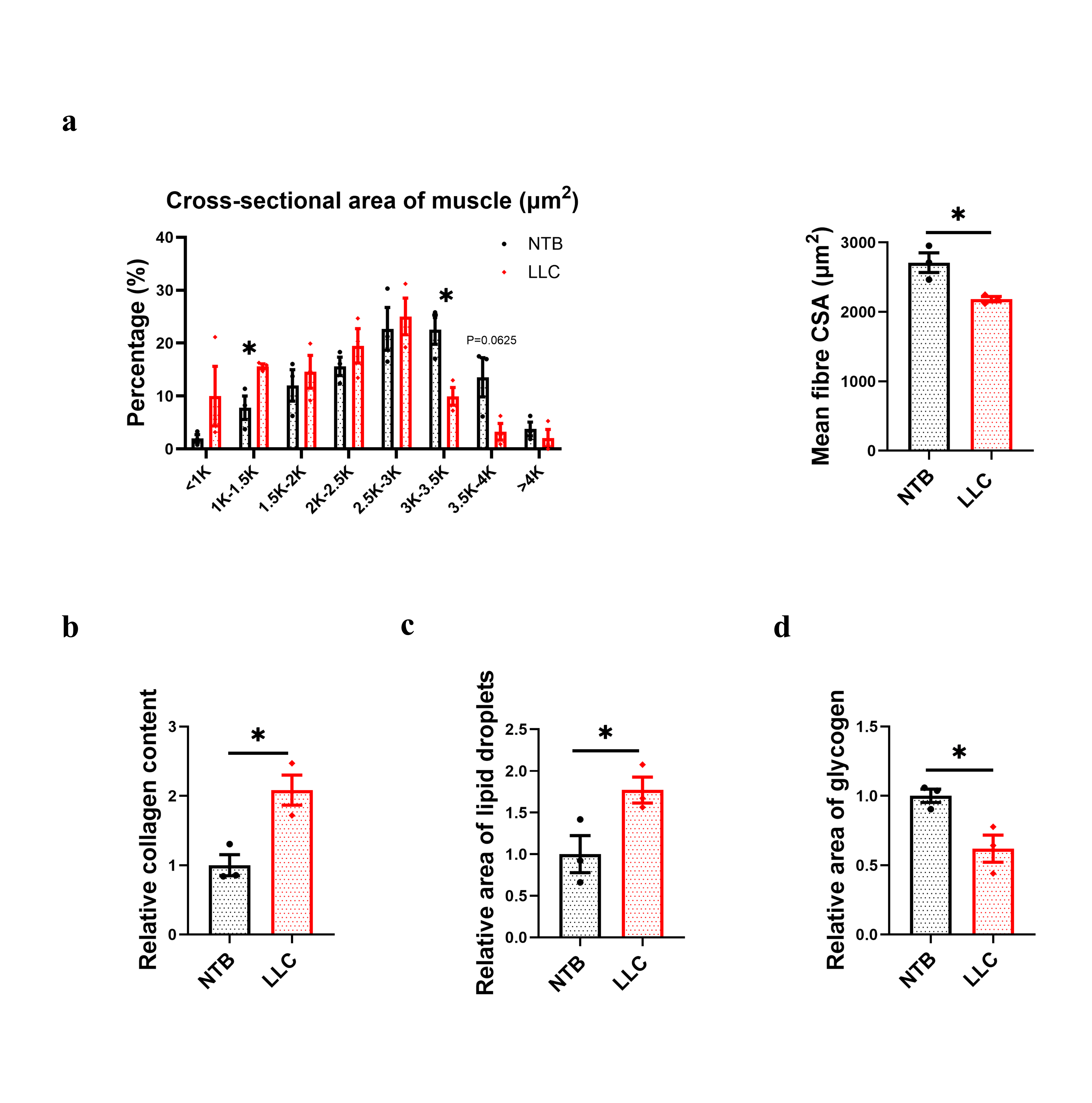

Supplement: Supplementary file 1 — Figure S1: The pathological results of quadriceps in NTB and LLC mice were quantitatively analysed using ImageJ. The fibre size distribution and the average CSA (n = 3/group). The relative collagen content (n = 3/group). The relative area of lipid droplets (n = 3/group). The relative area of glycogen (n = 3/group). [file JCSM-17-e70237-s004.tif]

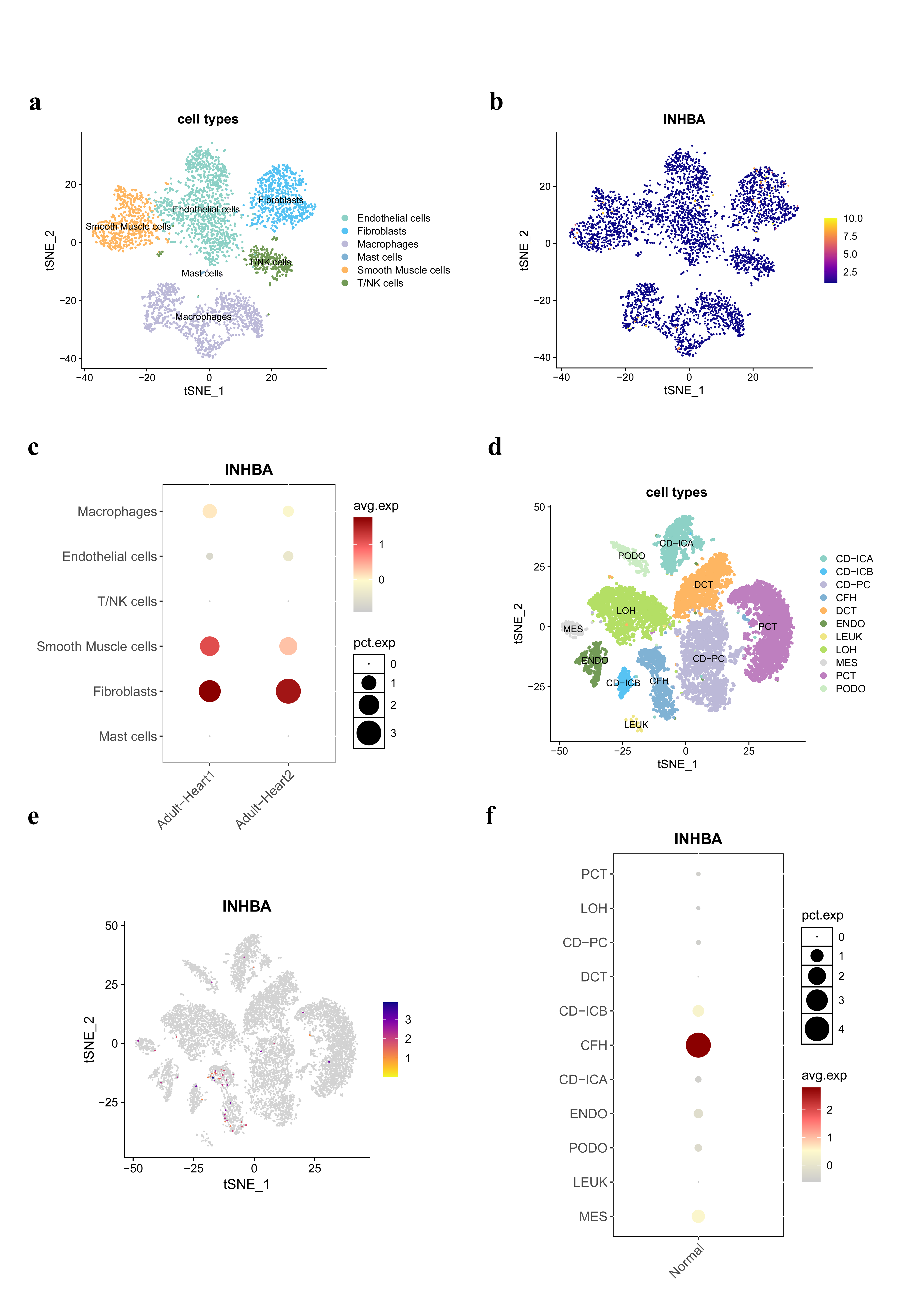

Supplement: Supplementary file 2 — Figure S2: jcsm70237‐sup‐0002‐Supplementary_Figure2.TIF. INHBA was widely expressed in normal human kidney and heart tissues. (a) t‐SNE projection of cells from human heart demonstrating 6 cell types. (b) t‐SNE plot showing the average expression level of INHBA in human heart cell types. (c) Dot plot showing the expression of INHBA in the human heart. (d) t‐SNE projection of cells from human kidney demonstrating 11 cell types. (e) t‐SNE plot showing the average expression level of INHBA in human kidney cell types. (f) Dot plot showing the expression of INHBA in the human kidney; PCT: proximal convoluted tubule; CFH: complement factor H; LOH: loop of Henle; DCT: distal convoluted tubule; CT: connecting tubule; CD: collecting duct; PC: principal cell; IC: intercalated cell; PODO: podocyte; ENDO: endothelium; MES: mesangial cell; LEUK: leukocyte. [file JCSM-17-e70237-s005.TIF]

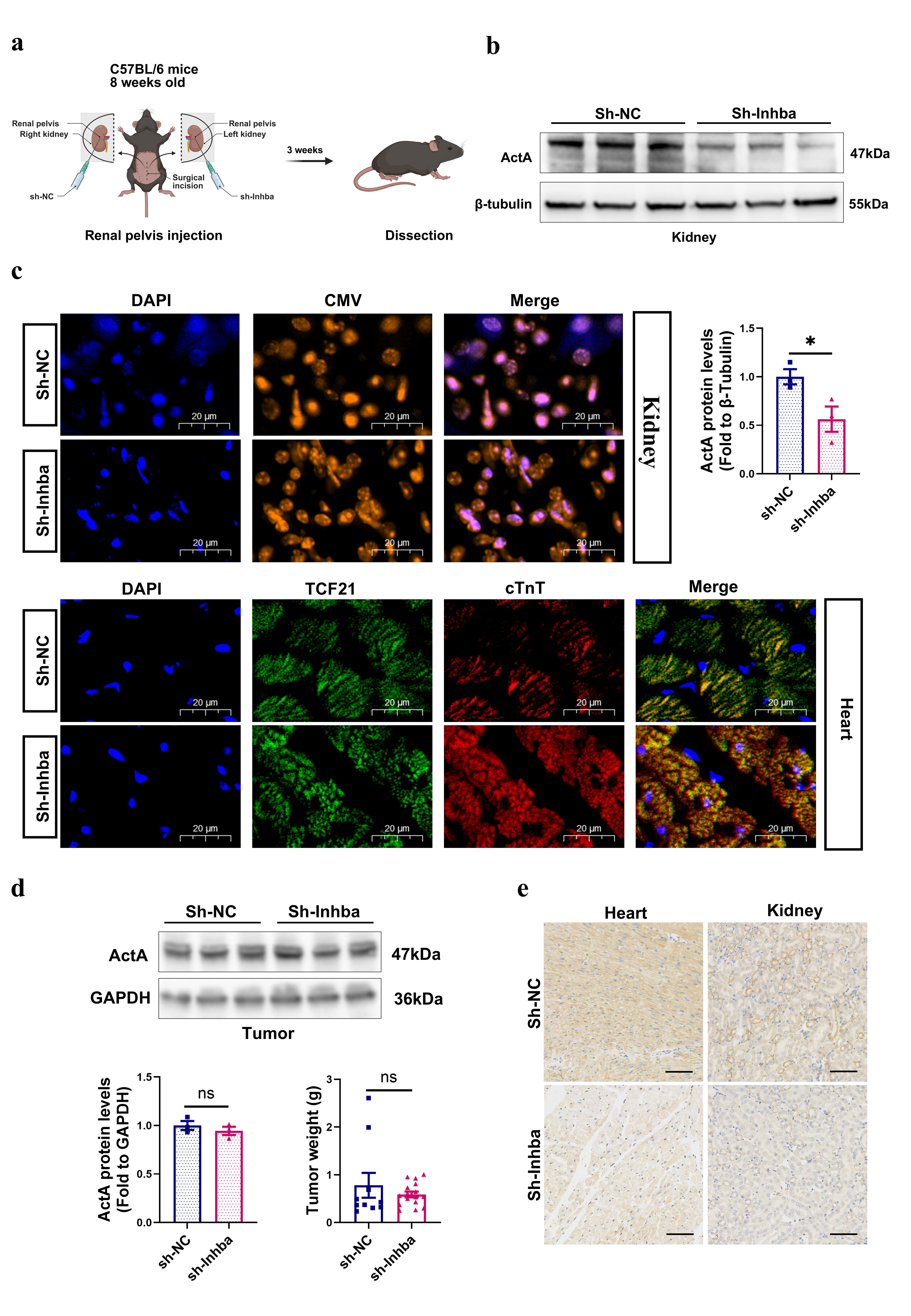

Supplement: Supplementary file 3 — Figure S3: Knocking down the expression of ActA in heart and kidney by injecting tissue‐specific AAV. (a) Schematic of study. (b) The protein levels of ActA in kidney were evaluated by western blot after AAV administration with the greatest ActA knockdown efficiency (n = 3/group). (c) The fluorescence images of heart and kidney. (d) The protein levels of ActA in tumour were evaluated by western blot and representative images were shown (n = 3/group); the tumours weight (g) (n = 10–16/group). (e) Representative IHC staining images of ActA in heart and kidney. Scale bar = 100 μm. [file JCSM-17-e70237-s002.TIF]

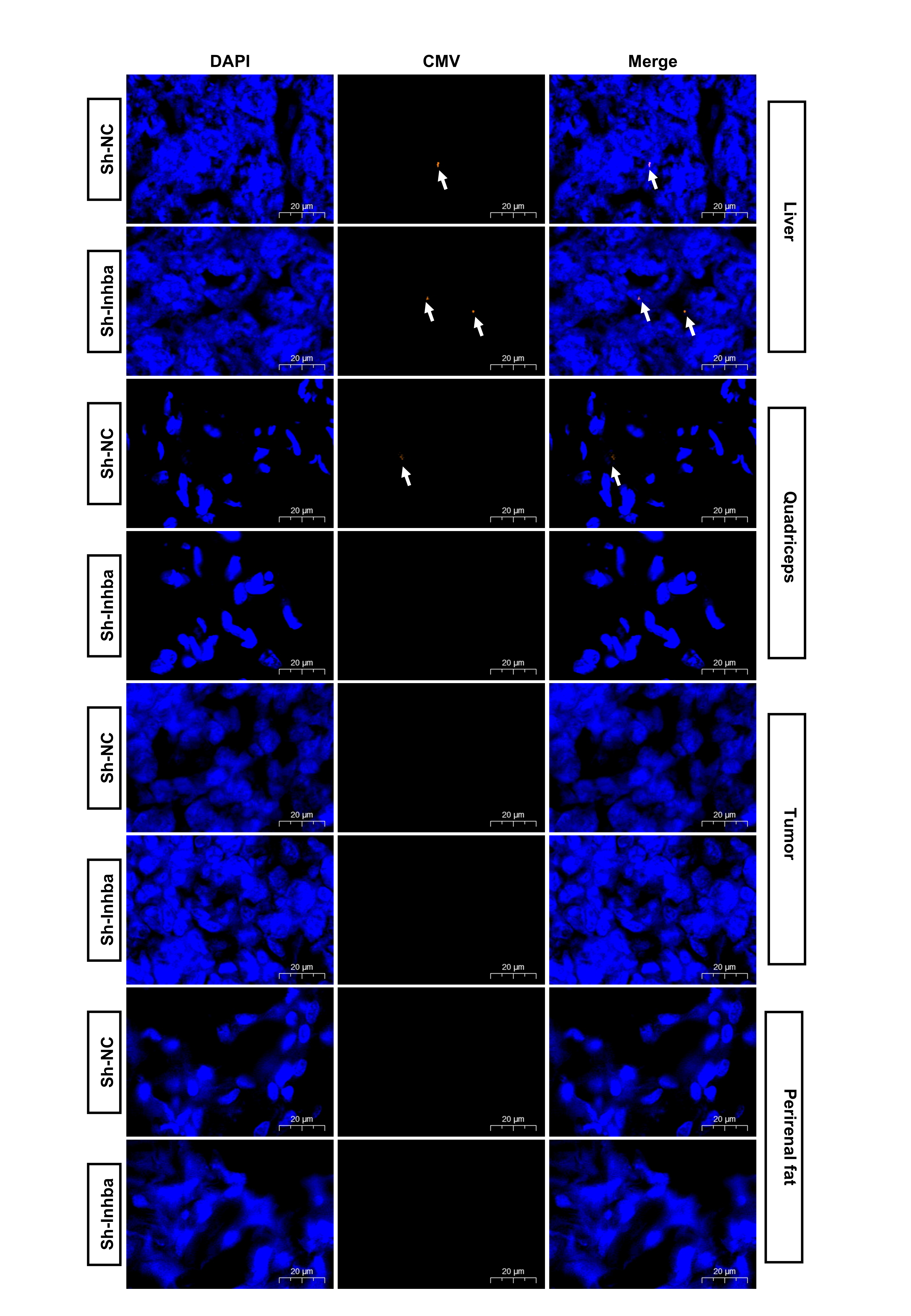

Supplement: Supplementary file 4 — Figure S4: The fluorescence images of liver, quadriceps, tumour and perirenal fat. [file JCSM-17-e70237-s001.TIF]
