# Supplementary material for: Downregulation of Organ‐Derived Activin A Attenuates Muscle Atrophy and Intramuscular Fat Infiltration in Cancer Cachexia Mice
Source: J Cachexia Sarcopenia Muscle. 2026 Mar 11;17(2):e70237. doi: 10.1002/jcsm.70237 (PMC12976578; doi:10.1002/jcsm.70237)
Supplement: Supplementary file 5 — Table S1: Oligonucleotides. Table S2: TaqMan primer pairs. [file JCSM-17-e70237-s003.docx]

**Table S1 Oligonucleotides**

| Gene | Oligo Sequence (5´- 3´) | |
| --- | --- | --- |
|  | **Forward** | **Reverse** |
| Actb | ACGGCCAGGTCATCACTATTG | TGGAAAAGAGCCTCAGGGC |
| MuRF1 | AGAAGTCGGGGGTCAGGGGACG | GGTCCATGATCACTTCATGGCGGCACGAGG |
| Atrogin-1 | ATGCACACTGGTGCAGAGAG | TGTAAGCACACAGGCAGGTC |
| TBP | GAAGCTGCGGTACAATTCCAG | CCCCTTGTACCCTTCACCAAT |
| ACC1 | CCTCCGTCAGCTCAGATACA | TTTACTAGGTGCAAGCCAGACA |
| FAS | GGTGTGGTGGGTTTGGTGAATTGT | TCACGAGGTCATGCTTTAGCACCT |
| SCD1 | CCGGAGACCCCTTAGATCGA | TAGCCTGTAAAAGATTTCTGCAAACC |
| CPT2 | CAACTCGTATACCCAAACCCAGTC | GTTCCCATCTTGATCGAGGACATC |
| CPT1B | TGGCTCATTTCCGGGACAAA | TGGTACAGGAACGCACAGTC |
| ACOX1 | GGTGGACCTCTGTCTTGTTCA | AAACCTTCAGGCCCAAGTGAG |

Table S1

**Table S2 TaqMan primer pairs**

| Genes | Sequence |
| --- | --- |
| Inhba | Mn00434338_ml |
| Actb | Mn02619580_gl |
| ACVR2A | Mn01331097_ml |
| ACVR2B | Mn00431664_ml |

Table S2
